# Chemokines as Potential Markers in Pediatric Renal Diseases

**DOI:** 10.1155/2014/278715

**Published:** 2014-02-17

**Authors:** Ana Cristina Simões e Silva, André Barreto Pereira, Mauro Martins Teixeira, Antônio Lúcio Teixeira

**Affiliations:** ^1^Unidade de Nefrologia Pediátrica, Departamento de Pediatria, Universidade Federal de Minas Gerais (UFMG), 30130-100 Belo Horizonte, MG, Brazil; ^2^Instituto Nacional de Ciência e Tecnologia em Medicina Molecular (INCT-MM), Faculdade de Medicina, UFMG, 30130-100 Belo Horizonte, MG, Brazil; ^3^Laboratório Interdisciplinar de Investigação Médica Faculdade de Medicina, UFMG, Avenida Alfredo Balena 190, 2nd Floor, Room No.281, 30130-100 Belo Horizonte, MG, Brazil; ^4^Departamento de Nefrologia, Santa Casa de Misericordia de Belo Horizonte, 30130-100 Belo Horizonte, MG, Brazil; ^5^Laboratório de Imunofarmacologia, Departamento de Bioquímica e Imunologia, Instituto de Ciências Biológicas, UFMG, 31270-901 Belo Horizonte, MG, Brazil

## Abstract

Glomerular diseases and obstructive uropathies are the two most frequent causes of chronic kidney disease (CKD) in children. Recently, biomarkers have become a focus of clinical research as potentially useful diagnostic tools in pediatric renal diseases. Among several putative biomarkers, chemokines emerge as promising molecules since they play relevant roles in the pathophysiology of pediatric renal diseases. The evaluation of these inflammatory mediators might help in the management of diverse renal diseases in children and the detection of patients at high risk to develop CKD. The aim of this paper is to revise general aspects of chemokines and the potential link between chemokines and the most common pediatric renal diseases by including experimental and clinical evidence.

## 1. Chemokines: General Concepts

Chemokines constitute a large family of low molecular-weight cytokines whose main action is the recruitment of leukocyte subsets under homeostatic conditions and inflammatio; the word “chemokine” is the contraction of the terms “chemotactic” and “cytokine” [1]. More specifically, leukocyte arrest during the rolling phase of its recruitment cascade is rapidly triggered by chemokines. After binding to specific seven transmembrane-domain G-protein-coupled receptors, chemokines regulate integrin-mediated adhesion among other effects [2].

To date, approximately 50 chemokines and 20 receptors have been described in humans [1, 3, 4]. Chemokines are divided into four families based on differences in structure and function, as shown in Table 1. In the context of leukocyte trafficking, chemokines can be functionally grouped as “homeostatic,” that is, chemokines constitutively expressed in organs; and “inflammatory,” that is, chemokines induced on inflamed sites [1, 3]. Although certain chemokines may be stored in granules of cells, such as platelets and mast cells, most chemokine expression is newly generated and released on demand at inflammatory sites [4].

With respect to renal diseases, there is much evidence that leukocyte infiltration is mediated by inflammatory chemokines released by various cell types [4, 5]. Infiltrating leukocytes produce chemokines that may amplify inflammatory responses in the kidney. Tubular epithelial cells can release inflammatory chemokines as CCL5/RANTES (regulated on activation, normal T expressed and secreted), CCL2/MCP-1 (monocyte chemotactic protein-1), CCL3/MIP-1*α* (macrophage inflammatory protein 1 alfa), CX3CL1/fractalkine, and CXCL8/IL8 (interleukin 8) [5]. Tubular epithelial cells are also targets for chemokines, since these cells respond to CCL2/MCP1 stimulation by releasing interleukin-6 (IL-6) and intracellular adhesion molecule-1 [6]. Messenger RNA of chemokines receptors can also be detected in podocytes and glomeruli [4].

There are several techniques to measure chemokine—protein or mRNA—in tissues and body fluids. For example, chemokines could be directly measured in renal tissue by immunohistochemical or immunofluorescent techniques or by evaluating their levels in supernatants of homogenized tissues. In patients with renal diseases, the direct exam of tissue samples would be ideal, since it may evaluate the affected organ. However, kidney biopsy is an aggressive procedure and could be harmful. On the other hand, enzyme-linked immunosorbent assay (ELISA) or flow cytometry based techniques are less invasive and more useful for clinical purposes by measuring the levels of chemokines in urine or blood samples [7–12]. Alternatively, chemokine mRNA can be measured by polymerase chain reaction or microarray in tissues or leukocytes of patients [8, 13, 14].

## 2. Chemokines in Renal Diseases

A number of studies have shown the relation between renal diseases and chemokines production [4–6]. Indeed, the measurement of urinary, plasma, and renal tissue levels of chemokines has been used to monitor and diagnose various renal diseases [7–12]. We cited below studies with chemokines most frequently associated to renal diseases.

CXCL8/IL-8 is a chemokine responsible for neutrophil infiltration into the urinary tract with an important role in acute pyelonephritis [15, 16]. In this regard, gene polymorphisms of this chemokine seem to increase the susceptibility to acute pyelonephritis [15]. For instance, the presence of the IL-8-251A allele in the genotype of children with urinary tract infection without vesicoureteral reflux has increased the risk of pyelonephritis [16].

CCL2/MCP-1 was the first CC chemokine to be discovered, acting as a potent chemotactic factor for monocytes, and has been one of the most studied biomarkers [6, 10, 13, 17–28]. Many studies have associated CCL2/MCP-1 with glomerulopathies [6, 13, 19–26] and with renal transplantation [10, 27, 28]. Wada and coworkers found significantly elevated urinary levels of this chemokine in adults with diabetic nephropathy, whereas serum levels remained similar to those of healthy volunteers [21]. Patients with active proteinuric forms of chronic glomerulonephritis have higher urine excretion of CCL2/MCP-1 than healthy controls [22]. In pediatric lupus nephritis, it was recently shown that increased urinary, but not plasma, CCL2/MCP-1 levels correlated with disease activity [20]. Taken together, these studies pointed to a potential role for CCL2/MCP-1 in glomerular inflammation. Concerning renal transplantation, it was previously reported that urinary levels of CCL2/MCP-1 were significantly higher in patients with acute rejection and a significant reduction of this chemokine was found in patients who responded to antirejection treatment [27]. In addition, increased urinary levels of CCL2/MCP-1 at 6 months after renal transplantation might predict renal allograft loss [28]. Urinary MCP-1 measurements may be an early marker of therapy responsiveness in patients with acute rejection.

CCL5/RANTES is a chemokine produced by human T lymphocytes at a “late” stage (3–5 days) after activation through their T-cell receptors. It is broadly chemoattractive for T lymphocytes, monocytes, natural killer cells, basophils, and eosinophils and can also activate immune cells. This chemokine is involved in AIDS, cancer, atherosclerosis, asthma, organ transplantation, and autoimmune diseases such as arthritis, diabetes, and glomerulonephritis [29].

In addition, some cytokines clearly interact with chemokines. As an example, Th-17 cells are a subset of Th cells, which produce IL-17A and IL-17F, members of the IL-17 cytokine family. These cytokines induce various cytokines/chemokines including CXCL8/IL8, IL-6, CCL2/MCP-1, TNF-*α*, IL-1*β*, G-CSF, and GM-CSF. IL-17 cytokines mediate the chemotaxis of neutrophils to sites of infection and upregulate the intercellular adhesion molecule-1 [30]. IL-17A has been extensively studied in pulmonary infections, asthma, cancer, ANCA associated vasculitis, glomerulonephritis, and renal transplantation [30–34].

## 3. Chemokines in Glomerular Diseases

In the last five years some, clinical studies measuring chemokines have been done in many forms of glomerular diseases such as IgA nephropathy, lupus nephritis, and idiopathic nephrotic syndrome (INS) including minimal change lesion (MCNS) and focal segmental glomerulosclerosis (FSGS).

IgA nephropathy is diagnosed by the predominance of IgA deposits in the glomerular mesangium and is present in around 13.8% of renal biopsies of children, being the second more frequent renal disease, and the mesangioproliferative glomerulonephritis is the most common histological presentation [35]. Several cytokines and chemokines (IL-1*β*, CCL2/MCP-1, IL-17, IFN-*γ*, IL-6, and IL-10) have been evaluated in IgA patients and some of them seemed to predict the outcome of the disease [36]. As an example, IL-17A was highly expressed in renal tubules of 34 patients with IgA nephropathy associated to lower renal function, greater proteinuria, and more severe tubulointerstitial damage than 29 patients with the same disease but without the increased expression of this marker [37].

Systemic lupus erythematosus (SLE) is a multisystemic autoimmune disease affecting predominantly women and 2/3 of cases occur in the first two decades of age. Lupus nephritis is a very common feature observed in 50–67% of children with SLE [38]. A wide range of cytokines and chemokines (IL-1, IL-9, IL-15, CXCL10/IP-10, CXCL2/MIP-1*α*, CCL5/RANTES, VEGF, IL-6, CXCL8/IL-8, IL-17, CCL2/MCP-1, CXCL3/MIP-1*β*, IL-2, IL-4, IL-5, IL-10, IL-12, IL-13, IFN-*γ*, and TNF-*α*) were tested for correlation with lupus activity by the Systemic Lupus Erythematosus Disease Activity Index (SLEDAI-1) [39]. The study showed that the chemokine CCL2/MCP-1 was the best biomarker of SLE activity. Regarding lupus nephritis, the serum B lymphocyte chemoattractant (CXCL13/BLC) was increased in 31 adult patients in comparison with 60 SLE patients without renal involvement and might be a surrogate available marker as well [40]. In a study of 60 consecutive childhood-onset SLE patients, serum IL-12 and TNF-*α* levels were significantly increased in patients with nephritis when compared to first-degree relatives and healthy controls, and TNF-*α* levels were significantly increased in patients with active disease [41]. Serum soluble receptor of TNF-*α* was elevated in 12 patients with active lupus nephritis compared with inactive SLE and healthy subjects and declined after clinical remission [42]. IL-17 and IL-23 were also elevated in serum and highly expressed in glomerulus of patients with lupus nephritis, suggesting the potential role of IL-23/Th17 axis on SLE with renal involvement [43, 44].

MCNS is the most common cause of nephrotic syndrome in children. The authors have proposed that this disease reflects a disorder of T-lymphocytes and some patients can progress to FSGS [45]. Araya and coworkers evaluated 23 patients with MCNS and 8 healthy controls and detected that MCNS patients have impaired T regulatory cells with low levels of IL-10 [8]. Woroniecki and coworkers studying 24 children with INS reported that urinary levels of the fibrogenic cytokine TGF-*β* might differentiate between FSGS and MCNS, but it seemed not to be a biomarker of steroid responsiveness [46]. Our research group measured plasma and urinary chemokines in 32 children with INS divided according to steroid responsiveness into 12 healthy controls [7]. We found increased levels of urinary IL8/CXCL8 in relapsed steroid resistant children when compared to steroid sensitive patients in remission, with a positive correlation with urinary protein levels [7]. These findings suggest that the renal release of the chemokine IL8/CXCL8 might be associated with changes in glomerular permeability [7]. More recently, by studying a group of pediatric patients at stages 2 to 4 of chronic kidney disease (CKD), we detected higher levels of urinary CCL2/MCP-1 in patients with FSGS than in cases of uropathies at the same stage of CKD [12]. In addition, urinary levels of CCL2/MCP-1 were positively correlated with serum total cholesterol and triglycerides concentrations [12]. This study supports the idea that differences in chemokine profile may be related to CKD etiology and other disease-associated alterations. Accordingly, Alzawa and coworkers measured CCL2/MCP-1 concentrations in the first morning urine samples obtained at the time of renal biopsy in 26 consecutive children with various types of glomerulonephritis, including lupus nephritis, IgA nephropathy, membranous nephropathy, acute GN, and thin basement membrane disease (served as a noninflammatory control) [47]. Urinary concentrations of MCP-1 showed a significant positive correlation with the degree of occult blood in urine and a significant inverse correlation with the estimated glomerular filtration rate. Furthermore, the urinary CCL2/MCP-1 concentration was significantly correlated with histological chronicity indices in patients with lupus nephritis and IgA nephropathy, supporting the hypothesis that the measurement of this chemokine may be useful as a noninvasive method for predicting the disease activity of glomerulonephritis in children [47].

**Figure 1:**
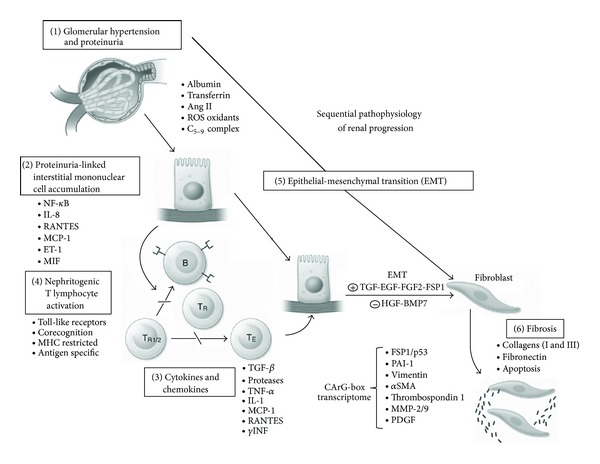
Sequential pathophysiological mechanisms related to the emergence of chronic kidney disease in glomerulopathies.

Figure 1 shows potential mechanisms that linked chemokines and the emergence of CKD in glomerular diseases.

## 4. Chemokines in Obstructive Uropathies

Congenital anomalies of the kidney and urinary tract (CAKUT) comprise a *spectrum* of malformations that occur at the level of the kidney (e.g., hypoplasia and dysplasia), collecting system (e.g., idiopathic hydronephrosis, ureteropelvic junction obstruction, and megaureter), bladder (e.g., ureterocele and vesicoureteral reflux), or urethra (e.g., posterior urethral valves) [48]. A variety of intrarenal factors lead to progressive interstitial and renal parenchyma fibrosis in patients with CAKUT, including growth factors, cytokines, chemokines and adhesion molecules, which are produced by the hydronephrotic kidney [49]. An altered renal expression of growth factors and cytokines modulates cell death by apoptosis or phenotypic transition of glomerular, tubular, and vascular cells. Mediators of cellular injury include hypoxia, ischemia, and reactive oxygen species, while fibroblasts undergo myofibroblast transformation with increased deposition of extracellular matrix. On the other hand, a number of endogenous antifibrotic counter-regulatory molecules have been identified, opening the possibility of enhancing the kidney's own defenses against progressive fibrosis [49, 50].

In this regard, chemokines like CCL2/MCP-1, CCL5/RANTES, macrophage inflammatory protein-2 (CXCL2/MIP-2), and *γ*-interferon-inducible protein (CXCL10/IP-10) have been evaluated in experimental hydronephrosis [49–53]. CCL2/MCP-1 is an inflammatory chemokine that attracts and activates monocytes, T-cells, and natural killer cells [4, 54]. Stephan and coworkers produced partial or complete ureteral obstruction in 28-day-old Wistar rats [55]. These authors found that CCL2/MCP-1 mRNA expression was moderately increased in partial ureteral obstruction, whereas kidneys without significant damage did not show any upregulation. The study qualifies CCL2/MCP-1 mRNA expression as a prognostic marker of partial ureteral obstruction [55]. In addition, Vielhauer and coworkers found an increased expression of the CC chemokines, CCL2/MCP-1 and CCL5/RANTES, at sites of progressive tubulointerstitial damage in murine obstructive nephropathy model [56]. An interstitial was also observed infiltration of macrophages and T lymphocytes, which differentially expressed the CCR2 receptors. These data suggest that CCR2- and CCR5-positive monocytes and CCR5-positive lymphocytes are attracted by locally released CCL2/MCP-1 and CCL5/RANTES, resulting in chronic interstitial inflammation [56]. Crisman and coworkers detected the expression of CCL2/MCP-1, CCL5/RANTES, and CXCL10/IP-10 at 1 day of unilateral ureteral obstruction in mice [57]. At 7 days, CCL5/RANTES became the most abundant chemokine in the obstructed kidney and the cortical tubular cells significantly contributed to this elevation [57]. The study of chemokines in hydronephrosis might provide new insights for the treatment or novel ways to blunt renal damage in obstructive nephropathy [58].

It should also be pointed that very few data about the role of chemokines in CAKUT were provided by clinical studies and the majority of them evaluated ureteropelvic junction obstruction (UPJO) and vesicoureteral reflux (VUR).

### 4.1. Ureteropelvic Junction Obstruction

UPJO is the most common cause of severe hydronephrosis in children [59–61]. UPJO is unilateral in 90% of cases and may result from intrinsic narrowing at the junction between ureter and renal pelvis or extrinsic compression by an accessory lower pole artery of the kidney [62]. The degrees of hydronephrosis vary among patients with UPJO. The histological changes may vary from the absence of abnormalities to renal dysplasia with glomerulosclerosis and extensive interstitial fibrosis and tubular atrophy [59–61]. The UPJO area is consistently inflamed and has varying degrees of fibrosis and muscular hypertrophy [59–61].

Postnatal differentiation between obstructive and nonobstructive hydronephrosis is quite difficult. Several studies have been made in patients with UPJO in order to find out noninvasive biomarkers to allow the diagnosis and treatment of these patients [58, 63, 64]. In this regard, cytokines, chemokines, and growth factors have been studied in UPJO [58, 63, 64]. Specifically for chemokines, the most relevant results were obtained with CCL2/MCP-1 [65–68].

Healthy children presented high expression of EGF mRNA in renal tissue, whereas CCL2/MCP-1 mRNA was normally undetectable. On the other hand, in UPJO patients, CCL2/MCP-1 gene expression was strikingly increased at the tubulointerstitial level, while the EGF gene expression was markedly reduced. The interstitial mononuclear cell infiltrate in UPJO patients was strictly correlated with the degree of tubulointerstitial damage [65, 66]. Accordingly, the urinary concentrations of EGF were reduced in UPJO patients, whereas the CCL2/MCP-1 levels were increased [65, 67]. After surgical correction, there was a significant reduction in urinary levels of CCL2/MCP-1 accompanied by a marked increase in EGF concentration. Therefore, these two biomarkers could be useful for the follow-up of obstructed patients [65]. In a prospective study, Madsen and coworkers reported that urinary concentrations of EGF and of CCL2/MCP-1 were significantly increased in preoperative samples collected in UPJO patients before surgical procedure in comparison to urine from healthy children [68]. At the same study, the concentrations of CCL2/MCP-1, CXCL1/MIP-1*α*, CXCL10/IP-10, and CCL5/RANTES were increased in urine from the obstructed kidney compared to urine from the contralateral nonobstructed kidney [68]. These urine samples were collected during the surgical procedure. One year after surgery, the concentrations of these chemokines were decreased to levels comparable to healthy controls [68].

Taranta-Janusz and coworkers compared obstructed prenatal hydronephrosis cases (who underwent surgery) with nonsurgically managed cases and with healthy subjects (control group) [69]. These authors found that urinary levels of CCL2/MCP-1 from voided urine before and after surgery and from the affected pelvis were significantly higher than non-surgically managed cases as well than control group [69]. Receiver operator characteristic (ROC) analyses revealed good diagnostic profile for urinary MCP-1 only in identifying children (<40%) with abnormal differential renal function (area under the curve (AUC) 0.862) and in detecting kidney injury in all examined children (AUC 0.704) [69]. The authors also studied the level of osteopontin (OPN) and CCL5/RANTES in urine samples. Urinary levels of OPN were significantly higher in surgical cases than in nonsurgically managed patients. Urinary levels of CCL5/RANTES were significantly higher in urine samples from affected pelvis collected during surgery than in voided urine before pyeloplasty. Three months after surgery, the urinary levels of these three biomarkers did not return to control values [69].

### 4.2. Vesicoureteral Reflux

VUR is a congenital anomaly that increases the risk of repeated pyelonephritis and, consequently, can result in renal scarring, renin-mediated hypertension, and, in some cases, renal insufficiency [70, 71]. VUR is a heterogeneous condition that can be primary or associated with multicystic kidney, hypodysplasic kidneys, renal agenesia, and renal or ureteral ectopia. Kidneys with reflux nephropathy have disjointed glomeruli from proximal tubules, interstitial infiltration with chronic inflammatory cells, and periglomerular fibrosis. Dysplasic feature is one of the characteristics of congenital reflux nephropathy. The main findings are areas of mesenchymal tissue containing primitive tubules [72].

The inflammatory process in VUR is ongoing despite the occurrence or not of urinary tract infection (UTI). The elevated urinary level of CXCL8/IL-8 in children with reflux and without UTI might contribute to reflux nephropathy [73–75]. Haraoka and coworkers have found a significant difference between urinary levels of CXCL8/IL-8 in children with and without renal scarring and in patients with and without VUR [73]. Merrikhi and coworkers also showed significantly higher levels of CXCL8/IL-8 in patients with RVU than in those without RVU [75]. This finding suggests that urinary CXCL8/IL-8 measurements could be useful to detect VUR patients with more pronounced renal damage and who need strict follow-up [75]. Galanakis and coworkers proposed the use of CXCL8/IL-8 as a biomarker for the diagnostis of VUR [74]. A ROC curve was constructed by plotting the sensitivity versus the specificity for different cutoff concentrations of CXCL8/IL-8/creatinine. With a cutoff concentration of urinary CXCL8/IL-8/creatinine at 5 pg/*μ*mol, the sensitivity of this marker in diagnosing VUR was 88%, the specificity 69%, the positive prognostic value 66%, and the negative prognostic value 89%. At higher cutoff concentrations, specificity of the marker increased, but sensitivity rapidly decreased [73]. Our research group has recently reported a correlation between high urinary levels of CXCL8/IL-8 and reduced glomerular filtration rate in CAKUT patients, suggesting that this chemokine might be associated with renal scarring and CKD [12].

**Figure 2:**
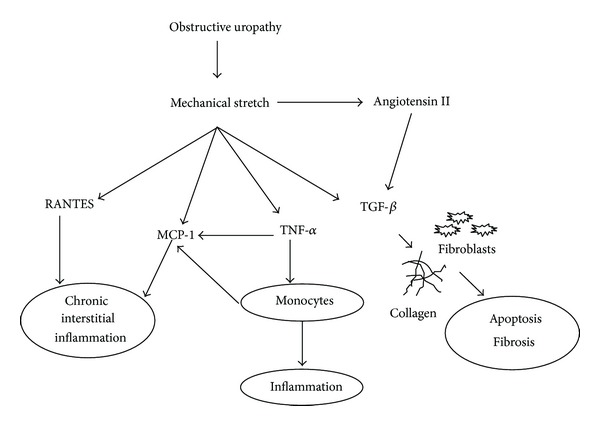
Schematic view of chemokine and fibrogenic factors release at renal tissue in obstructive uropathies.

Figure 2 proposes a connection between chemokine release and CKD in patients with obstructive uropathies.

## 5. Concluding Remarks

New diagnostic approaches to and alternative therapies for pediatric renal diseases are clearly necessary. In this context, research into biomarkers has reached great importance. In 2001, an NIH working group standardized the definition of a biomarker as “a characteristic that is objectively measured and evaluated as an indicator of normal biological processes, pathogenic processes, or pharmacologic responses to a therapeutic intervention” [76]. In this context, understanding the pathogenic role of chemokines on pediatric renal diseases is of interest not only as new prognostic markers but also as alternative therapeutic targets.

We believed that spot urine measurements of chemokines might become useful in pediatric renal diseases even at clinical practice. For instance, CCL2/MCP-1 and CXCL8/IL-8 are the chemokines more commonly associated with pediatric renal diseases [7, 11, 12, 15, 16, 19, 20, 46, 47, 55, 58, 65–69, 73–75].

In regard to glomerular diseases, high urinary levels of CCL2/MCP-1 have been frequently related to FSGS [12, 22, 23, 45, 46], lupus nephritis [20, 39, 47], and IgA nephropathy [24, 36, 47]. Therefore, high urinary levels of CCL2/MCP-1 might indicate the presence and severity of glomerular injury in spite of the etiology. There is also evidence of a potential link between monocyte recruitment and dyslipidemia in pediatric patients with CKD due to FSGS [12]. Concerning CXCL8/IL-8, urinary levels of this chemokine have been positively correlated with proteinuria in patients with INS, suggesting a role in glomerular permeability changes [7, 8]. However, we do not know yet if these biomarkers are related to disease prognosis or to pharmacologic responses to therapeutic interventions.

In pediatric patients with CAKUT, the chemokine CCL2/MCP-1 has been associated with urinary tract obstruction in UPJO [65–68], whereas high urinary levels of CXCL8/IL-8 were found in VUR [73–75]. This chemokine has been also correlated to the presence of renal scarring [73] renal function deterioration in CAKUT patients [12]. Based on these results, it should be further investigated if urinary measurements of CCL2/MCP-1 would help detecting obstructive uropathies and if high urine levels of CXCL8/IL-8 would indicate the presence of renal scarring in VUR.

Yet, in spite of great advances in our knowledge about the pathophysiological mechanism linking the chemokines to renal diseases, much remains to be elucidated. Furthermore, the usefulness of chemokine measurements in clinical practice still needs to be established.

## Figures and Tables

**Table 1:** The families of chemokines described in humans (IUPHAR nomenclature/original name).

Chemokine family	Structure	Function	Main members
CC chemokines.	The first two cysteine residues are adjacent to each other.	Attraction of mononuclear cells to sites of chronic inflammation.	CCL2/MCP-1 CCL3/MIP-1*α* CCL5/RANTES

CXC chemokines subfamily ELR (+).	The first two cysteine residues are separated by a single aminoacid with a glutamic acid-leucine-arginine (ELR) motif near the N terminal of the molecule.	Attraction of polymorphonuclear leukocytes to sites of acute inflammation.	CXCL8/IL-8

CXC chemokines subfamily ELR (−).	The first two cysteine residues are separated by a single aminoacid without ELR motif.	IFN-*γ* inducible chemokines, which are involved in the recruitment of Th1 lymphocytes.	CXCL9/MIG CXCL10/IP-10 CXCL11/I-TAC

CX3C chemokines.	The first two cysteine residues are separated by three amino acids.	Chemokines expressed on activated endothelial cells responsible for leucocyte adhesion and migration.	CX3CL1/fractalkine

XC chemokines.	With a single cysteine residue.	Attraction of certain subsets of T-cells and natural killer cells	XCL1/lymphotactin-*α* XCL2/lymphotactin-*β*

CCL2/MCP-1: monocyte chemotactic protein-1; CCL3/MIP-1*α*: macrophage inflammatory protein 1 alfa; CCL5/RANTES: regulated on activation, normal T expressed and secreted; CXCL8/IL-8: interleukin-8; CXCL9/MIG: monokine induced by gamma interferon; CXCL10/IP-10: interferon gamma-induced protein 10; CXCL11/I-TAC: interferon-inducible T-cell alpha chemoattractant.
